# Prognostic value of alkaline phosphatase in hormone-sensitive prostate cancer: a systematic review and meta-analysis

**DOI:** 10.1007/s10147-019-01578-9

**Published:** 2019-11-25

**Authors:** Keiichiro Mori, Florian Janisch, Mehdi Kardoust Parizi, Hadi Mostafaei, Ivan Lysenko, Dmitry V. Enikeev, Shoji Kimura, Shin Egawa, Shahrokh F. Shariat

**Affiliations:** 1 Department of Urology, Medical University of Vienna, Währinger Gürtel 18-20, 1090 Vienna, Austria; 2 Department of Urology, The Jikei University School of Medicine, Tokyo, Japan; 3 Department of Urology, Medical University of Hamburg, Hamburg, Germany; 4 Department of Urology, Shariati Hospital, Tehran University of Medical Sciences, Teheran, Iran; 5 Research Center for Evidence Based Medicine, Tabriz University of Medical Sciences, Tabriz, Iran; 6 Department of Urology, Weill Cornell Medical College, New York, NY USA; 7 Department of Urology, University of Texas Southwestern, Dallas, TX USA; 8 Karl Landsteiner Institute of Urology and Andrology, Vienna, Austria; 9 Department of Urology, Second Faculty of Medicine, Charles University, Prague, Czech Republic; 10 Institute for Urology and Reproductive Health, I.M. Sechenov First Moscow State Medical University, Moscow, Russia

**Keywords:** Alkaline phosphatase (ALP), Hormone-sensitive prostate cancer (HSPC), Meta-analysis

## Abstract

**Purpose:**

To assess the prognostic value of alkaline phosphatase in patients with hormone-sensitive prostate cancer.

**Methods:**

A systematic review and meta-analysis was performed using the PUBMED, Web of Science, Cochrane Library, and Scopus in April 2019 according to the Preferred Reporting Items for Systematic Review and Meta-analysis statement. Studies were deemed eligible if they compared hormone-sensitive prostate cancer patients with high vs. low alkaline phosphatase to determine its predictive value for overall survival, cancer-specific survival, and progression-free survival. We performed a formal meta-analysis of these outcomes.

**Results:**

42 articles with 7938 patients were included in the systematic review and 28 studies with 5849 patients for the qualitative assessment. High alkaline phosphatase was associated with worse overall survival (pooled HR 1.72; 95% CI 1.37−2.14) and progression-free survival (pooled HR 1.30; 95% CI 1.10−1.54). In subgroup analyses of patients with “high-volume” and “low-volume”, alkaline phosphatase was associated with the overall survival (pooled HR 1.41; 95% CI 1.21−1.64 and pooled HR 1.64; 95% CI, 1.06−2.52, respectively).

**Conclusions:**

In this meta-analysis, elevated serum levels of alkaline phosphatase were associated with an increased risk of overall mortality and disease progression in patients with hormone-sensitive prostate cancer. In contrast, those were not associated with an increased risk of cancer-specific mortality. Alkaline phosphatase was independently associated with overall survival in both patients with “high-volume” and “low-volume” hormone-sensitive prostate cancer. Alkaline phosphatase may be useful for being integrated into prognostic tools that help guide treatment strategy, thereby facilitating the shared decision making process.

## Introduction

Prostate cancer (PC) is not only the most common solid cancer, but also the second most common cause of cancer-related death in men [1]. Following the results of the CHAARTED trial and the LATITUDE trial, the treatment of patients with metastatic hormone-sensitive prostate cancer (HSPC) has changed substantially in the recent years [2, 3]. However, systemic therapy based on androgen deprivation remains the standard primary treatment strategy in patients with metastatic HSPC. Despite adequate therapy, the disease eventually progresses to a castration-resistant prostate cancer (CRPC) [4]. To improve PC outcomes, prognostic tools have been developed to help in the daily clinical decision making and patient counselling [5–8]. These tools include standard clinical features and biomarkers [9], such as alkaline phosphatase (ALP) in patients with CRPC, but not yet in patients with HSPC.

ALP is a glycoprotein derived from bones, liver, kidney, or placenta that has been shown to be elevated and of prognostic value for various malignancies [10–13]. In PC, ALP has been shown to be of prognostic value in CRPC-reflecting disease outcome, independent of therapy [14]. In patients with CRPC, high-baseline ALP levels have been shown to be associated with worse outcomes, including skeletal complications and decreased survival [15–17]. Moreover, elevated ALP was also been shown to be correlated with the extent of metastatic bone disease [17, 18]. Serum ALP is deemed a simple and inexpensive test that could serve as an objective prognostic parameter that helps improve daily oncologic clinical practice, plan follow-up, and counsel regarding outcomes, thus facilitating the shared decision making process with the patient. Unfortunately, to date, the prognostic value of ALP in HSPC remains insufficiently investigated.

The aim of the current study was to summarize the available data to test the hypothesis that ALP has a strong prognostic value for oncologic outcomes in HSPC patients. To this end, we performed a systematic review and a meta-analysis.

## Materials and methods

### Search strategy

This systematic review and meta-analysis was performed according to the Preferred Reporting Items for Systematic Reviews and Meta-analyses (PRISMA) statement [19]. We searched the electronic databases PUBMED, Web of Science, Cochrane Library and Scopus on April 2019, investigating the prognostic value of ALP in HSPC.

After the first screening based on study title and abstract, all papers were assessed based on full text and excluded with reasons when inappropriate; a further check of the appropriateness of the papers based on full text revision which was performed after the data extraction. Two investigators carried out this process independently. Disagreements were resolved by a consensus meeting with a third investigator. The following keywords were used in our search strategy: (prostate cancer OR prostate carcinoma OR prostate tumor OR prostatic carcinoma OR prostatic cancer OR prostatic tumor NOT resistant) AND (Alkaline Phosphatase OR ALP) AND (survival OR outcome OR prognostic OR mortality OR progression OR recurrence OR OS OR CSS OR PFS OR RFS OR MFS). The primary outcome of interest was overall survival (OS) and secondary outcomes were cancer-specific survival (CSS) and progression-free survival (PFS).

### Inclusion criteria and exclusion criteria

Studies were included if they investigated whether patients with high ALP treated for HSPC (patients) who had received systemic therapy (intervention) as compared to those who had low ALP (comparison) to assess the independent predictive value of ALP on OS, CSS, and PFS (outcome) utilizing multivariate Cox regression analysis (study design) in nonrandomized observational, or randomize or cohort studies. We excluded reviews, letters, editorials, meeting abstracts, replies from author, case reports, and articles not published in English. In case of duplicate publications, either the higher quality or the most recent publication was selected. References of included manuscripts were scanned for additional studies of interest.

### Data extraction

Two investigators independently extracted the information from the included articles. The information contained the following characteristics: first author’s name, publication year, recruitment country, period of patient recruitment, number of patients, age, study design, disease stage, therapy type, oncological outcome, follow-up duration, conclusion, and ALP cut-off. Subsequently, the hazard ratios (HR) and 95% confidence intervals (CI) of ALP associated with each of the outcomes were retrieved. The HRs were extracted from the multivariate analyses. All discrepancies regarding data extraction were resolved by consensus with a third investigator.

### Quality assessment

The Newcastle–Ottawa Scale (NOS) was used to assess the quality of the included studies according to the Cochrane Handbook for systematic reviews of interventions for included non-randomized studies [20, 21]. The scale focuses on the three factors: Selection (1−4), Comparability (1−2) and Exposure (1−3). The total score ranges from 0 (lowest) to 9 (highest). The main confounders were identified as the important prognostic factors of OS, CSS, and PFS. The presence of confounders was determined by consensus and review of the literature. We identified as “high-quality” choices those with scores more than 6.

### Statistical analyses

We performed a forest plot to assess the HRs from the multivariate analyses of individual studies and obtained a summary HR of the value of ALP on OS, CSS, and PFS. Disease progression includes symptomatic or radiographic or biochemical progression in this analysis. Studies with Kaplan–Meier log-rank, univariate Cox proportional hazard regression, or general logistic regression analyses were not considered for the meta-analysis. In case there were only HR and *P* value, we calculated 95% CI [22, 23]. We also performed subgroup analyses in HSPC patients with “high-volume” and “low-volume” disease. We classified as low-volume (lesions < 4 sites and within pelvis–vertebral column) or high-volume disease (lesions ≥ 4 sites and at least one lesion beyond the pelvis–vertebral column) according to the CHAARTED classification [2]. Again, of all the HSPC patients from the studies providing information on EOD scores or Soloway scores, those with EOD scores 2 or higher or those with Soloway scores 2 or higher were defined as high-volume disease [24]. With high-volume disease thus defined, all studies in which those with high-volume disease accounted for 60% or more or less than 60% of all patients were included for the current analysis as “high-volume disease” and “low-volume disease” studies, respectively.

Heterogeneity among the outcomes of the included studies in this meta-analysis was evaluated using Cochrane *Q* test and *I*^2^ statistic. Significant heterogeneity was indicated by a *P* < 0.05 in Cochrane *Q* tests and a ratio > 50% in *I*^2^ statistics, which led to the use of random-effect models. We used fixed effect models for calculation of pooled HRs for non-heterogeneous results [25–27]. Publication bias was assessed by funnel plots. Statistical analyses were performed using Stata/MP 14.2 (Stata Corp., College Station, TX); statistical significance level was set at *P* < 0.05.

## Results

### Study selection and characteristics

**Fig. 1:**
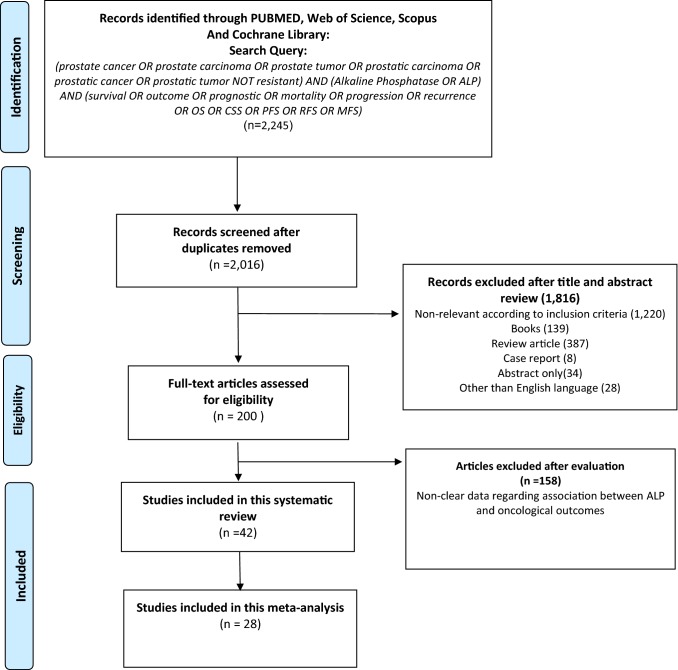
The Preferred Reporting Items for Systematic Reviews and Meta-analyses (PRISMA) flow chart for article selection process to analyze the prognostic value of alkaline phosphatase (ALP) in hormone-sensitive prostate cancer (HSPC) and oncological outcomes

Our initial search identified 2245 records. After removal of duplicates, 2016 remained (Fig. 1). After screening of the titles and abstracts, 1816 articles were excluded. Then we assessed 200 full texts for further selection. After selection, 42 articles with 7938 patients were included in the systematic review and 28 studies with 5849 patients for qualitative meta-analysis [28–69]. The baseline characteristics of the 42 studies are outlined in Table 1. All included studies were published between 1995 and 2019 with 15 being from Europe, and 27 from Asia. Median age ranged from 63 to 77 years, 10 studies included non-metastatic HSPC. Studies were heterogeneous regarding cut-off value for ALP ranging from 67 to 620 for OS, from 115 to 683.4 for CSS, and from 114.56 to 400 for PFS; follow-up ranged from 14.4 to 156 months.

**Table 1:** Study characteristics of 42 studies

Author	Year	Country	Recruitment period	N	Age	Design	Metastasis	Treatment	Outcome	Follow up (month)	Conclusion	Cut off (IU/L)	NOS
Reynard	1995	UK	1986–1990	85	71	P	M1	E	OS	NR	P	UNL	6
Vasalainen	1995	Finland	1971–1992	188	71.5	R	M0, M1	E	OS	156	P	UNL	6
Stokkel	1997	Netherland	1990–1995	124	71	R	M0	E, P, R	OS, PFS	41	N	NR	6
Furuya	1998	Japan	1986–1993	139	73.6	R	M1	E	CSS	36.9	N	NR	7
Akimoto	1999	Japan	NR	48	71.8	R	M1	E	CSS	32	N	NR	7
Nakashima	2000	Japan	NR	114	NR	R	M1	E	OS	NR	N	620	6
Kwak	2002	Korea	1991–1997	151	67.7	R	M0, M1	E	OS	39	P	115	7
Pelger	2002	Netherland	NR	233	75	R	M0, M1	E	PFS	NR	P	NR	7
Furuya	2003	Japan	1990–1999	59	72.9	R	M1	E	CSS	25.3	N	UNL	6
Noguchi	2003	Japan	1994–2000	56	72	R	M1	E	CSS	32	P	467	5
Yashi	2003	Japan	NR	70	72	R	M1	E	PFS	27.4	N	400	7
Jung	2004	Germany	1998–2001	117	66	R	M0, M1	E	OS	36.1	N	129	6
Brasso	2006	Denmark	1993–1996	153	72	P	M1	E	OS	59	P	Continuous	7
Salminen	2006	Finland	NR	84	67	R	M0, M1	E	OS	52	P	227	6
Saito	2007	Japan	1992–2004	241	72.3	R	M1	NR	OS	31	P	500	7
Robinson	2008	Sweden	1992–1997	697	72.8	P	M1	E	CSS	37	P	UNL	7
Jeong	2009	Korea	1987–1995	295	69.7	R	M0, M1	E	OS, CSS	39	P	115	7
Lein	2009	Germany	2002–2005	117	NR	P	M1	E,C	PFS	NR	N	NR	6
Mikkola	2009	Finland	1990–1994	142	72	P	M1	E	OS	NR	P	180	6
Kamiya	2010	Japan	2002–2008	58	69	R	M1	E	CSS	35	N	683.4	6
yamada	2010	Japan	1998–2006	104	74	R	M1	E	CSS	43	N	UNL	7
Jung	2011	Germany	2002–2005	52	68	R	M1	E	OS	49	N	67	8
Miyamoto	2011	Japan	1992–2002	94	72.5	R	M1	E	OS	38.8	P	220	7
He	2012	China	1997–2009	115	72	R	M1	E	OS	26.8	P	NR	6
Tsuchiya	2013	Japan	1980–2008	215	72	R	M1	E	CSS	37	P	350	7
Nozawa	2014	Japan	2008–2010	52	72	P	M1	E	OS, PFS	41.6	P	300	5
Gravis	2015	France	2004–2008	385	63	P	M1	E, C	OS	58.3	P	UNL	6
Koo	2015	Korea	2002–2012	248	NR	R	M1	E	CSS, PFS	39.9	P	200	7
Mohammed	2015	Saudi Arabia	2011–2015	71	72	R	M1	NR	CSS	14.4	P	NR	6
Kato	2016	Japan	2002–2012	150	73	R	M1	E	OS	38	N	398	7
Klaff	2016	Sweden	1992–1997	319	NR	P	M1	E	OS	112.5	N	1.25xUNL	7
Klaff	2016	Sweden	1992–1997	483	NR	P	M1	E	OS	63.3	P	1.25xUNL	7
Lv	2016	China	2009–2014	168	72	R	M1	E	PFS	22	P	114.56	6
Pan	2016	China	2009–2012	155	NR	P	M1	E	OS, PFS	38	N	220	7
Peng	2016	China	1997–2012	113	64	R	M1	E	OS	41	P	150	7
Josefsson	2017	Sweden	2012–2015	40	77	P	M1	E	PFS	NR	P	Continuous	6
Wang	2017	China	2004–2015	438	70	R	M0	P	PFS	52	N	Continuous	7
Buelens	2018	Belgium	2014–2018	113	70	P	M1	E,C	OS	20	P	UNL	6
Okamoto	2018	Japan	2005–2017	339	72	R	M1	E	OS, CSS, PFS	26	N	322	7
Sato	2018	Japan	2000–2015	60	72	R	M1	E	OS, PFS	34	P	UNL	7
Zhao	2018	China	2011–2016	449	NR	R	M1	E	OS, PFS	50	P	UNL	7
Miyake	2019	Japan	2010–2017	437	NR	R	M1	E	OS	46.5	P	400	7
Shimodaira	2019	Japan	1999–2012	167	74.8	R	M0, M1	E	CSS	54.3	P	350	6

*C* chemotherapy, *CSS* cancer-specific survival, *E* endocrine therapy, *N* (outcome): negative, *NOS* Newcastle–Ottawa Scale, *NR* not reported, *OS* overall survival, *P* (design) prospective, *P* (outcome) positive, *P* (treatment): prostatectomy, *PFS* progression-free survival, *R* (design) retrospective, *R* (treatment) radiotherapy, *UNL* upper normal limit

### Meta-analysis

#### Association of ALP with OS in HSPC

**Fig. 2:**
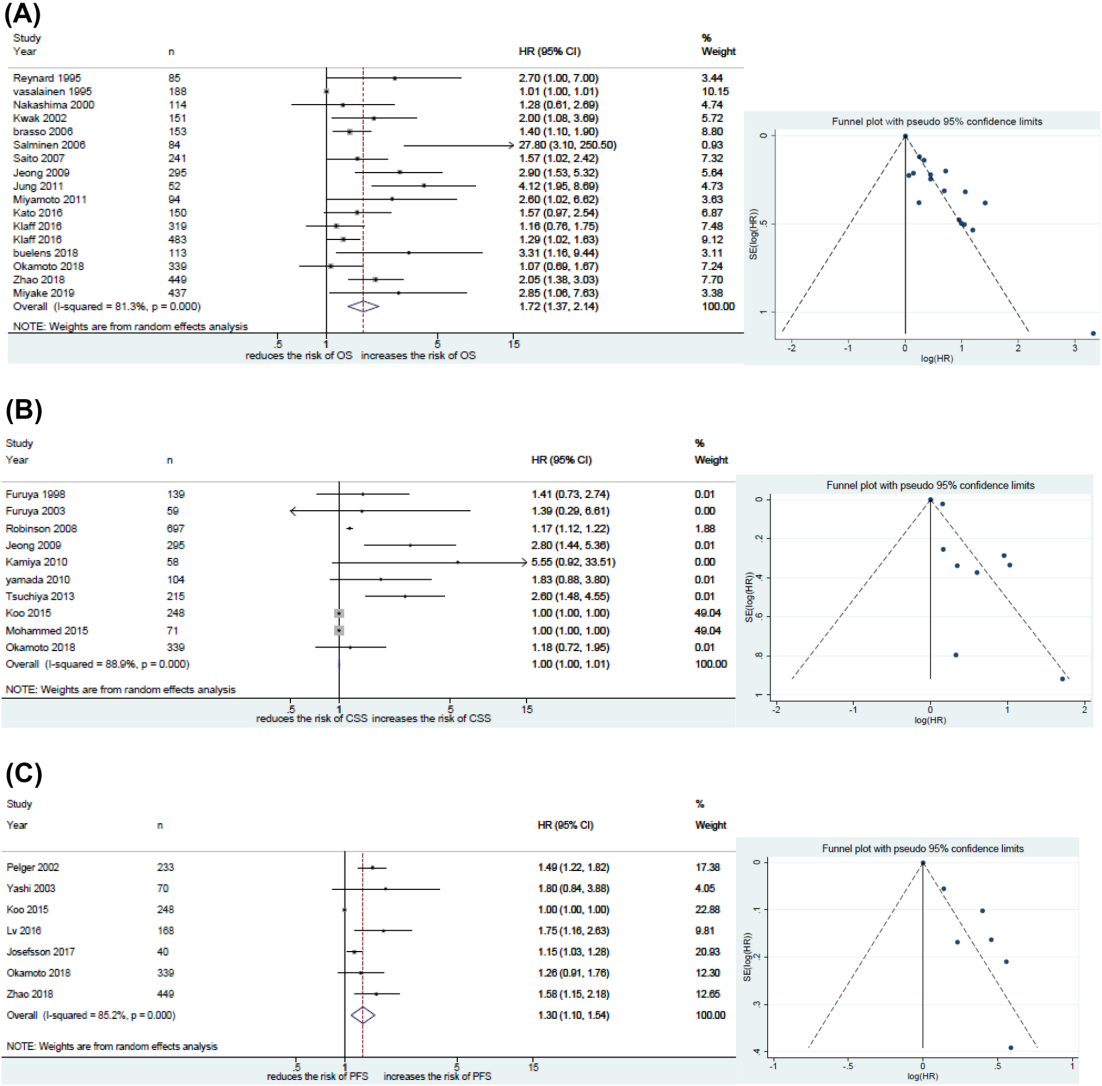
Forest and funnel plots showing the association of alkaline phosphatase (ALP) with oncologic outcomes in hormone-sensitive prostate cancer (HSPC): **a** overall survival **b** cancer specific surivival (C) progression free survival

Sixteen studies including 3747 patients provided data on the association of ALP with OS in HSPC. The forest plot (Fig. 2a) showed that ALP was significantly associated with OS in HSPC (pooled HR 1.72; 95% CI 1.37 − 2.14; *z* = 4.76). The Cochrane *Q* test (*χ*^2^ = 85.73; *P* = 0.000) and *I*^2^ test (*I*^2^ = 81.3%) showed significant heterogeneity. The funnel plot identified nine studies over the pseudo 95% CI (Fig. 2a).

#### Association of ALP with CSS in HSPC

Ten studies including 2225 patients provided data on the association of ALP with CSS in HSPC. The forest plot (Fig. 2b) showed that ALP was not significantly associated with CSS in HSPC (pooled HR 1.00; 95% CI 1.00–1.01; *z* = 1.55). The Cochrane *Q* test (*χ*^2^ = 80.97; *P* = 0.000) and *I*^2^ test (*I*^2^ = 88.9%) showed significant heterogeneity. The funnel plot identified four studies over the pseudo 95% CI (Fig. 2b).

#### Association of ALP with PFS in HSPC

Seven studies including 1547 patients provided data on the association of ALP with PFS in HSPC. The forest plot (Fig. 2c) showed that ALP was significantly associated with PFS in HSPC (pooled HR 1.30; 95% CI 1.10−1.54; *z* = 3.04). The Cochrane *Q* test (*χ*^2^ = 40.49; *P* = 0.000) and *I*^2^ test (*I*^2^ = 85.2%) showed significant heterogeneity. The funnel plot identified four studies over the pseudo 95% CI (Fig. 2c).

#### Association of ALP with OS in HSPC with “high volume”

**Fig. 3:**
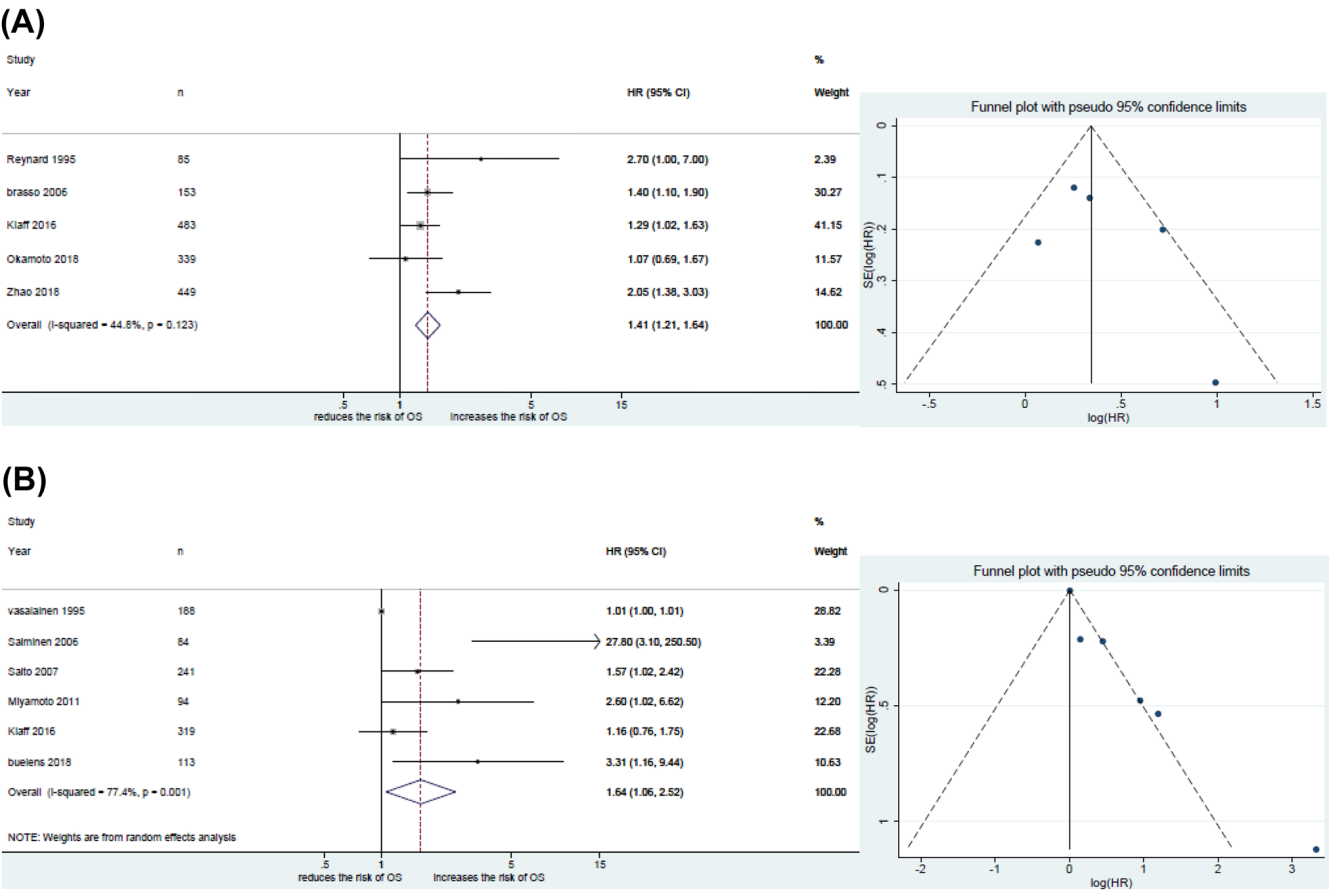
Forest and funnel plots showing the association of alkaline phosphatase (ALP) with oncologic outcomes: **a** overall survival in hormone-sensitive prostate cancer (HSPC) with “high-volume” disease **b** overall survival in hormone-sensitive prostate cancer (HSPC) with “low-volume” disease

Five studies including 1509 patients provided data on the association of ALP with OS in HSPC with “high-volume” disease. The forest plot (Fig. 3a) showed that ALP was significantly associated with OS in HSPC with “high-volume” disease (pooled HR 1.41; 95% CI 1.21−1.64; *z* = 4.47). The Cochrane *Q* test (*χ*^2^ = 7.25; *P* = 0.123) and *I*^2^ test (I^2^ = 44.8%) showed no significant heterogeneity. The funnel plot identified no studies over the pseudo 95% CI (Fig. 3a).

#### Association of ALP with OS in HSPC with “low volume”

Six studies including 1039 patients provided data on the association of ALP with OS in HSPC with “low-volume” disease. The forest plot (Fig. 3b) showed that ALP was significantly associated with OS in HSPC with “low-volume” disease (pooled HR 1.64; 95% CI 1.06−2.52; *z* = 2.25). The Cochrane *Q* test (*χ*^2^ = 22.10; *P* = 0.001) and *I*^2^ test (*I*^2^ = 77.4%) showed significant heterogeneity. The funnel plot identified three studies over the pseudo 95% CI (Fig. 3b).

## Discussion

In this systematic review and meta-analysis, we investigated the prognostic value of ALP in HSPC by assessing its impact on PFS, CSS, and OS. We found that the HSPC patients with elevated ALP have significantly worse OS and PFS compared to their counterparts with normal ALP levels. In other words, pre-treatment ALP values may be a useful biomarker in the choice of treatment, even in early metastatic PC.

The prognostic value of ALP has been shown in various solid malignancies with bone metastasis [11–13]. However, while there is a biological rationale underlying this association, the exact mechanism remains unclear. A potential explanation is that when cancer starts to metastasize, ALP reflects bone turnover, osteoblast activity, and osteoid formation in the adjacent bone tissues [70]. Thus, ALP may be an indicator of bone metastatic tumor load. Accordingly, ALP has been shown to be elevated in cancer patients with bone metastasis, as the current literature shows, ALP is already among the biomarkers included in the tools used for prognosticating outcomes in CRPC patients [5–8].

Interestingly, ALP was significantly associated with worse OS in metastatic HSPC patients not only with “high-volume” disease, but also with “low-volume” disease, suggesting that ALP is an indirect sensitive measure of metastatic tumor burden which could not be captured by conventional imaging. It is likely that the elevated ALP reflects micro metastases despite negative findings on conventional imaging. Although few studies have assessed this patient subgroup, ALP could be used to select patients who may benefit more from intensive therapy such as upfront docetaxel or abiraterone in addition to standard androgen deprivation therapy. Moreover, ALP could also be used as a response/monitoring marker for these therapies as well as bone-targeting therapies such as bisphosphonate.

Despite showing a strong association of ALP with mortality and progression in HSPC patients, this systematic review and meta-analysis has some limitations. There is a reporting bias, as some studies with negative results may not have been published. Further, many included studies were retrospective, leading to a patient selection bias. Second, unknown pretreatment conditions (i.e., physical conditions, comorbidities, obstructive jaundice, bone disease, hyperthyroidism and hepatitis, medication, and life-style habits) could have altered ALP values leading to a systematic bias. Third, heterogeneity was detected for OS, CSS, and PFS analyses limiting the value of these results. Although the random effect model takes into account the heterogeneity among studies, the conclusions should be interpreted with caution. Fourth, there is no established cut-off value for ALP among the included studies; most investigators chose the cut-off based on the statistical methods assessing for the highest sensitivity and specificity, using the upper limit of normal, or using literature predefined ALP cut-offs. Only three studies investigated ALP as a continuous variable. Regardless of these limitations, ALP is a fast and readily available biomarker. Well-designed prospective studies with longer follow-up are needed to validate the prognostic value of ALP and its potential value in risk stratification of patients with HSPC using clinical decision-analytical tools.

## Conclusions

In this meta-analysis, high serum ALP was associated with an increased risk of overall mortality and disease progression in patients with HSPC. In contrast, high serum ALP was not associated with an increased risk of cancer-specific mortality. Furthermore, ALP was an independent risk factor for OS in HSPC patients with both “high-volume” and “low-volume” metastatic disease. ALP may be useful for clinical decision making regarding treatment selection, as well as for patient counselling. However, considering the limitations including heterogeneity, the conclusions should be interpreted with caution.
